# Supplementary material for: Learning curve and surgical outcome of robotic assisted colorectal surgery with ERAS program
Source: Sci Rep. 2022 Nov 29;12:20566. doi: 10.1038/s41598-022-24665-w (PMC9709162; doi:10.1038/s41598-022-24665-w)
Supplement: Supplementary file 1 — Supplementary Information 1. [file 41598_2022_24665_MOESM1_ESM.zip › LSC Raw data-Ñ[▒K/128/O_ñw╝╨░O▒Kñσ.pdf]

正式摘要(電子簽章：000000218436737)

請選擇...reporter

臺中榮民總醫院

Operation Note

[redacted]

- 手術日期：2021/06/07
- 手術醫師：VS 蔣鋒帆，CR 林陳彥，R 古朋育，R 洪浚嚴
- Anesthesia : GE
- Position : Supine
- Aseptic agents : Betadine Aqua

Diagnosis

- Pre-operative diagnosis : R/O T-colon tumor with adenomatous polyposis syndrome
- Post-operative diagnosis : s/p total colectomy

Surgical Indication

- For removing the suspicious tumor lesions

Operation

- 1. Conventional total colectomy with side to end, ileorectal anastomosis (IRA)
  # Initially, laparocopic was attempted. It was later shifted to conventional, opening method.
  2. Intraoperative colonoscopy : (-)
  3. High ligation (+) : At IMA root. IMV and left colic artery were also ligated. Ileocolic vessels were also ligated. Distal middle colic vessels were ligated.
  4. EndoGIA (60-4.1) X3 + EndoGIA (60-3.5) X1 + EEA (25 mm) were applied.
  5. One 12 Fr. penrose drainge tube was placed from LLQ of abdomen and inserted into the pelvic area
  6. The colon was taken down from the splenic flexure site and hepatic flexure site

Finding

- 1. Severely adhesion was noted between the S-colon, appendix and the nearby abdominal wall
  2. Redundant omentum and severely adhesion was also presented over the splenic flexure area. The operation was then shifted to open method.
  3. Polyposis was noted over the whole colon. A suspicious, probably malignant lesion over T-colon was presented.
  4. Suspicious, hardended mass over appendix. May be post inflammation related ? Tumor related ?

Procedure

- 1. General endotracheal anesthesia was induced and then the patient was placed in modified Trendelenburg lithotomy position. Preoperative antibiotics were given. A Foley catheter and a nasogastric tube were placed. The abdomen was prepared and draped in standard sterile fashion.
  2. A small vertical incision was made right next to the umbilicus and a 12 mm trocar was inserted into the peritoneal cavity by open method. Peumoperitoneum was established with CO2 inflation. Laparoscopy was inserted and then another 12 mm trocar was inserted over RLQ. Extra two 5 mm trochars were inserted over RUQ and LLQ of abdomen. Another 12mm trocar was inserted over LUQ of abdomen. The operative findings were mentioned as above.
  3. Initially, lysis was performed for better surgical field.
  4. The small bowel was inspected and retracted to the right by grasper. The sigmoid colon was then held by grasper and the peritoneum overlying the mesentery was then scored with electrocautery. The inferior mesentery artery was identified, ligated below the left colic artery with endoclips, and

- transected.
  5. The arcade vessels to the sigmoid colon were also divided. Distal descending colon and upper rectum were mobilized. Totally three linear staplers (EndoGIA 60-4.1) were used to divide the rectum.
  6. Due to the severe adhesion and redundant omentum status, the procedure was shifted to open method.
  7. An upper vertical midline incision was made. This was deepened through the subcutaneous tissues and hemostasis was achieved with electrocautery.
  8. The abdomen was explored and wound retractor (Alexis) was set up.
  9. The avascular line of Toldt along the left paracolic gutter was incised from the sigmoid colon to splenic flexure as possible
  10. The lesser sac was approached and the omentum was resected carefully via Harmonic scaple. The gastrocolic omentum was divided as far to the left as possible until the splenic flexure was no longer tethered. The distal branches of the left middle colic vessels were also identified and then resected and ligated with double 2-O silks
  11. The peritoneum overlying the mesentery was then scored with electrocautery. IMV was also ligated with 2-O silk.
  12. Using Harmonic scaple, the colon was partial freed from its peritoneal attachments along the line of Toldt from the cecum.
  13. We approached the cecum area and the ileocolic artery area was identified. It was resected and ligated with double tie method. A suspicious, hardended mass was palpated over the appendix area.

  14. The distal branch of the right middle colic vessels were identified and ligated with double tie method.
  15. The hepatic flexure part until the middle T-colon area was carefully freed after the remaining small vessels were ligated.
  16. Finally, cecum, ascending colon, hepatic flexure and T-colon were fully mobilized.
  17. The field was isolated with moist pad and the bowel opened and sized. It was determined that a 25 mm circular staple could be accommodated.
  18. A small incision was made near the end of the terminal ileum, which then gentally extended to allow the anvil to be inserted. The anvil was penetrated from the lateral side of the terminal ileum. (For later side to end approach)
  20. Then the anvil was fixed with mersilk sutures. The residule terminal end of the terminal ileum was sealed and resected via firing one staple of EndoGIA (60-3.8). The whole specimen was then taken down.
  21. The anal canal was irrigated with distil water and then the circular stapler was introduced through the anus and pierced through the mid-portion of the stapler line.
  22. The proximal bowel with anvil in place was engaged with the circular stapler and the stapler was then fired. Two complete donuts were obtained.
  23. The anastomosis was tested for leaks with irrigation solution and air introduced through the rectum and was found to be patent and intact.
  24. The possible bleeder was then checked thoroughly. Massive warm saline was applied to irrigate the abdomen cavity.
  25. One 12 Fr. penrose drainge tube was placed from LLQ of abdomen and inserted into the pelvic area
  26. The ADHESION BARRIER FILM was then placed below the central abdominal wound.
  27. The fascia of midline wound was closed with interrupted sutures of Num. 1 Vicryl. The fascia of the 12 mm trochar was also closed with 1-0 Vicryl.
  28. After irrigation, the skin were closed with interrupted 3-0 nylon, and the wound was covered with Medipore.
  29. The patient tolerated the procedure well and was brought to the POR in stable condition.
- Estimated blood loss: minimum
- 器械數量: 正確
- 紗布數量: 正確

Image

- PAINT
  - abdomen

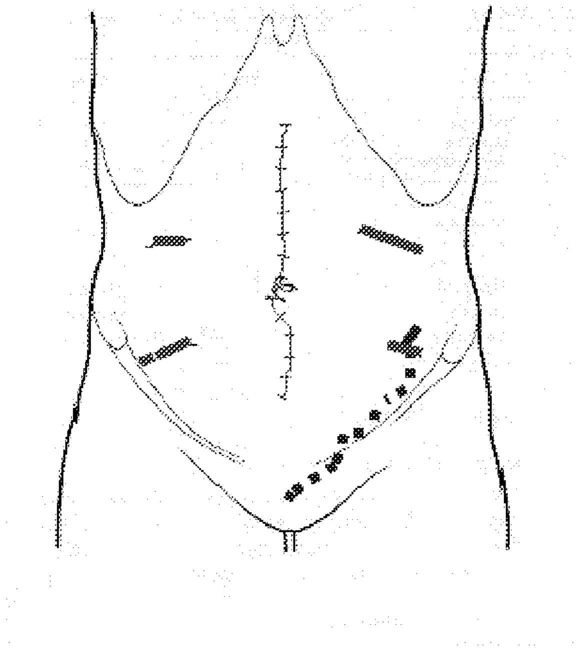

- colon & rectum

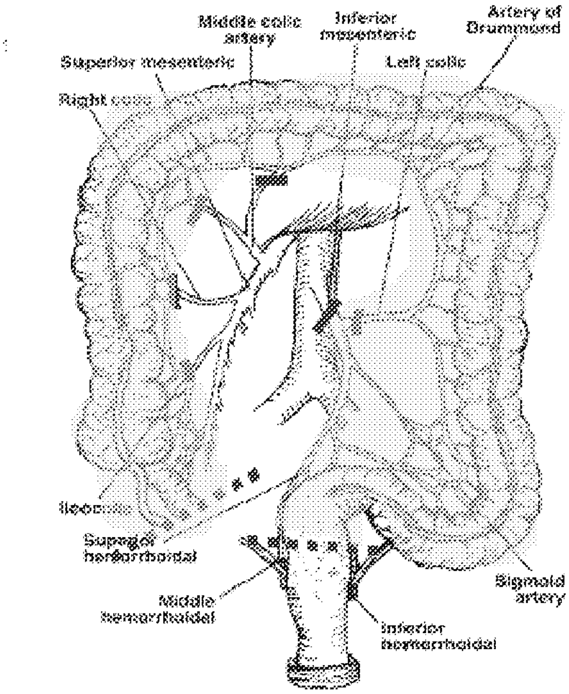

- The resected specimen

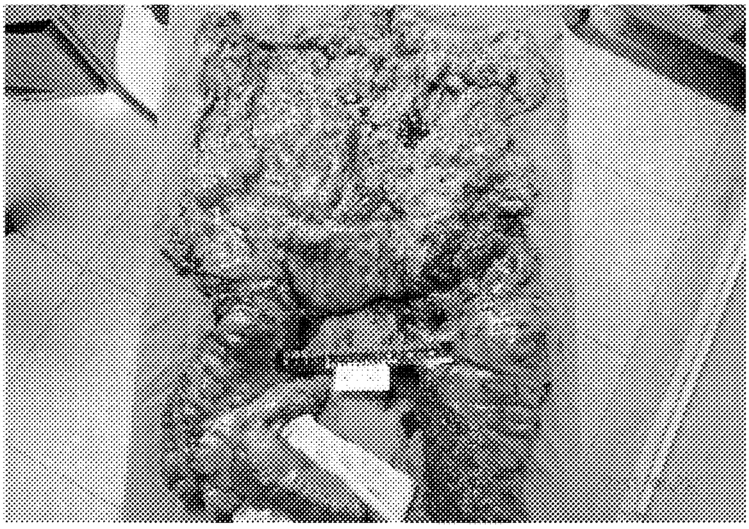

Polyposis condition

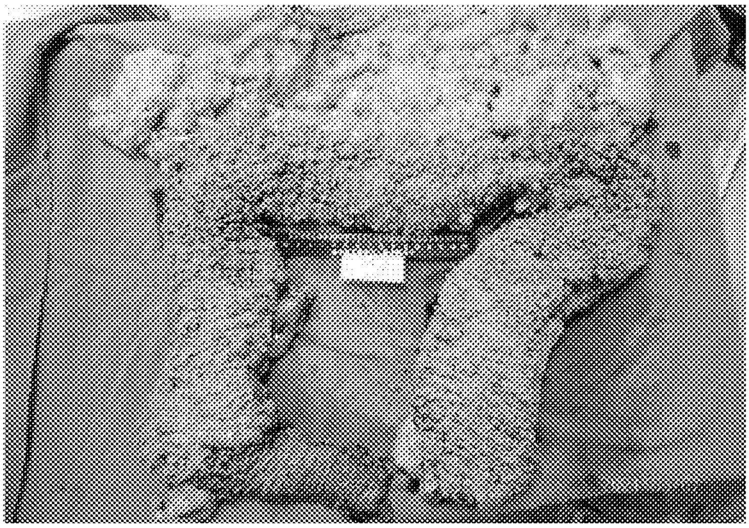

The suspicious lesion over appendix area

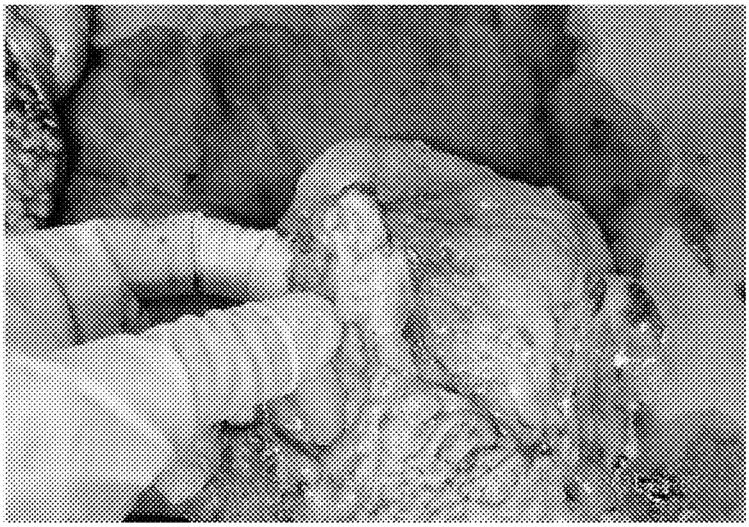

主任 2062F蔣鋒帆 / 醫師 2301H林陳彥

正式摘要(電子簽章：000000218436737)

請選擇...reporter

doctorRank:B
